# Supplementary material for: Development of a Probability-Based In Vitro Eye Irritation Screening Platform
Source: Bioengineering (Basel). 2024 Mar 26;11(4):315. doi: 10.3390/bioengineering11040315 (PMC11047661; doi:10.3390/bioengineering11040315)
Supplement: Supplementary file 1 [file bioengineering-11-00315-s001.zip › bioengineering-2845071-supplementary.pdf]

Supplementary Information

Development of a probability-based in vitro eye irritation screening platform

Seep Arora[#1], Anna Goralczyk[#1], Sujana Andra[^1], John Soon Yew Lim[2], Yi-Chin Toh[* 1,3,4,5]

[1]Department of Biomedical Engineering, National University of Singapore, Singapore 117583

[2]A*STAR Microscopy Platform, 61 Biopolis Drive, #06-20 Proteos, Singapore 138673

[3]School of Mechanical, Medical and Process Engineering, Queensland University of Technology, Brisbane, QLD 4000, Australia

[4]Centre for Biomedical Technologies, Queensland University of Technology, Kelvin Grove, QLD 4059, Australia

[5]ARC Training Centre for Cell and Tissue Engineering Technologies, Kelvin Grove, QLD 4059, Australia

Supplementary Figures

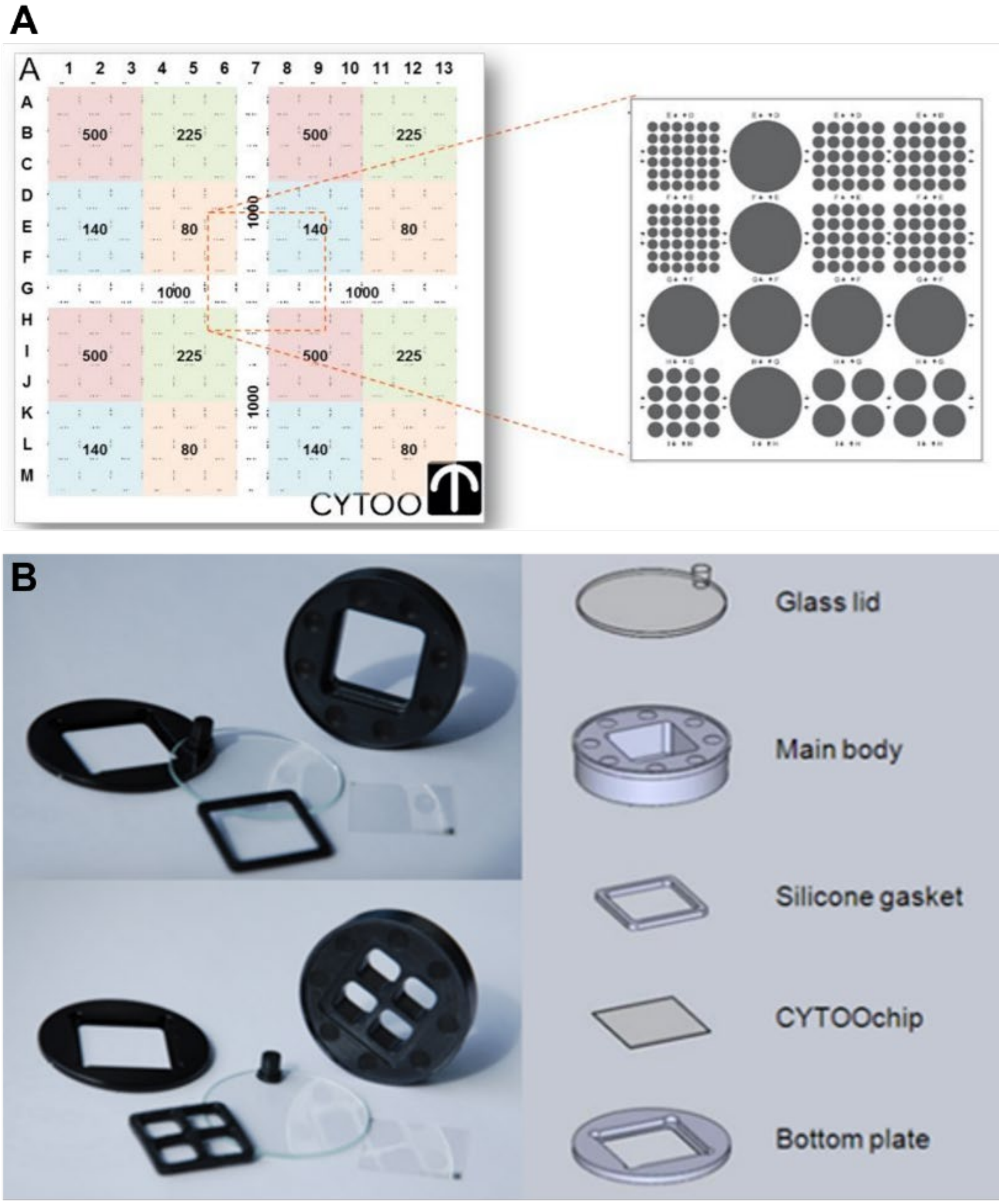

SI Figure S1. Commercial cell micropatterning platform. (A) CYTOO chip design. Different diameters (from 500 down to 80 μm) are organized over the chip in 4 identical quadrants. Each quadrant is again divided into 4 zones and each disc diameter width is arrayed over 3x3 blocks. The blocks separating the 4 quadrants feature the discs of 1000 μm (one disc per block) (B) Components of CYTOO chamber to partition CYTOO chip into 4 wells. Photos are harnessed from the supplier: https://cytoo.com/

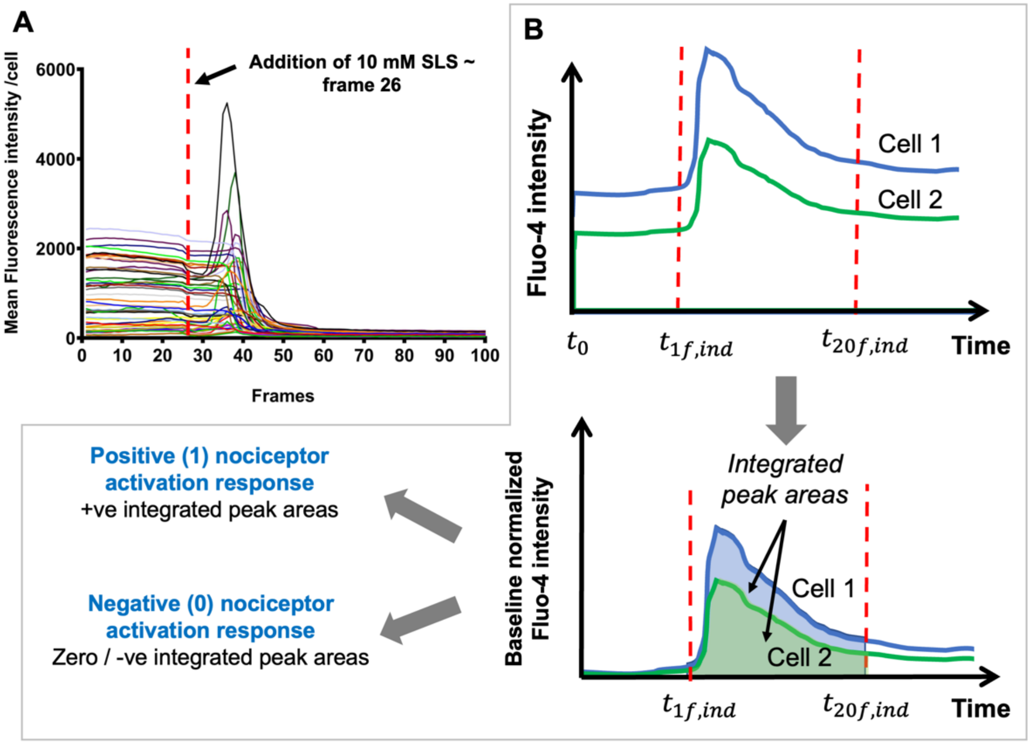

SI Figure S2. Quantification and binarization of TRPV1 activation based on Ca^{2+} responses elicited by SLS and SMA. (A) Change in Fluo-4 fluorescence intensity per cell over time triggered by 10 mM SLS in primary hCECs. (B) Process flow to binarize Ca2+ influx response.
